# Leukocyte Telomere Length and Its Polygenic Risk Score in Post‐Stroke Cognitive Impairment: Evidence From a Multicenter Cohort Study

**DOI:** 10.1002/brb3.71435

**Published:** 2026-05-14

**Authors:** Kunying Zhao, Yanfeng Shi, Hongyi Yan, Hongyu Zhou, Jiejie Li, Si Cheng, Zhe Xu, Yuesong Pan, Zixiao Li, Xia Meng, Hao Li, Xiaoling Liao, Jing Jing, Yongjun Wang

**Affiliations:** ^1^ Department of Neurology, Beijing Tiantan Hospital Capital Medical University Beijing China; ^2^ Department of Neurology Beijing Fengtai Hospital Beijing China; ^3^ China National Clinical Research Center For Neurological Diseases Beijing China

**Keywords:** aging, post‐stroke cognitive impairment, polygenic risk score, stroke, telomere length

## Abstract

**Background:**

Post‐stroke cognitive impairment (PSCI) is a frequent and disabling consequence of ischemic stroke. Leukocyte telomere length (LTL), a biomarker of systemic biological aging, has been implicated in cognitive outcomes, but evidence in stroke populations remains limited. We investigated whether baseline LTL and an LTL polygenic risk score (LTL‐PRS) are associated with PSCI risk in a large Chinese stroke cohort.

**Methods:**

We analyzed patients with ischemic stroke or transient ischemic attack (TIA) from the Third China National Stroke Registry–impairment of cognition and sleep (CNSR‐III–ICONS) sub study. Baseline LTL was estimated from whole‐genome sequencing (WGS) using TelSeq. An 18‐SNP LTL‐PRS (East‐Asian weights) was z‐standardized. Cognitive status at 12 months was assessed using the Montreal Cognitive Assessment (MoCA); PSCI was defined as an age‐, sex‐, education‐adjusted norm‐referenced threshold (z score ≤ −1.5). Fifty‐one participants missing education required for the adjusted PSCI definition were excluded, leaving 923 for analysis. Multivariable logistic regression tested associations of LTL and LTL–PRS with PSCI, with exploratory interaction and stratified analyses by age, sex, and stroke subtype.

**Results:**

PSCI occurred in 191 of 923 patients (20.7%). Neither continuous LTL nor LTL–PRS was associated with PSCI overall. Exploratory analyses provided nominal evidence of heterogeneity by age (p‐interaction = 0.038) and stroke subtype (p‐interaction = 0.01), whereas evidence for sex interaction was inconclusive (p‐interaction = 0.07). Associations with the MoCA executive function domain were weak and sensitive to covariate adjustment.

**Conclusions:**

Baseline telomere metrics showed, at most, modest and context‐dependent associations with PSCI. Neither LTL nor this limited‐variant LTL–PRS demonstrated overall prognostic utility, highlighting the need for integrated, longitudinal multi‐omic approaches for individualized cognitive risk prediction after stroke.

## Graphical abstract

In a multicenter Chinese ischemic stroke/TIA cohort (n = 923), baseline leukocyte telomere length (LTL) and an 18‐SNP LTL polygenic risk score (LTL‐PRS) were not independently associated with 12‐month norm‐referenced MoCA‐defined post‐stroke cognitive impairment after multivariable adjustment; nominal heterogeneity by age and TOAST subtype was exploratory.

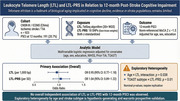

## 1 Introduction

Post‐stroke cognitive impairment (PSCI) affects a substantial proportion of stroke survivors, undermining independence, quality of life, and long‐term prognosis (El Husseini et al. 2023; Rost et al. 2022). Despite guideline attention, clinically actionable tools for early and accurate PSCI risk stratification remain limited (El Husseini et al. 2023; Rost et al. 2022; Filler et al. 2024). Against this backdrop, aging biology provides a unifying framework linking cerebrovascular disease to neurocognitive decline and motivates evaluation of aging‐related biomarkers for prognostication (Filler et al. 2024; Blackburn et al. 2015).

Telomere attrition is a canonical molecular hallmark of aging and integrates cumulative oxidative and inflammatory stress (Rossiello et al. 2022; López‐Otín et al. 2023). Leukocyte telomere length (LTL) is readily assayed and has been associated with ischemic stroke risk (Li et al. 2018; Yetim et al. 2021; Ding et al. 2012; Haycock et al. 2014) as well as cognitive decline and dementia in population cohorts, although reported effect sizes are generally small and heterogeneous (Hägg et al. 2017; Gampawar et al. 2022; Liu et al. 2023). In stroke populations, shorter LTL has also been linked to post‐stroke mortality and subsequent cognitive decline, raising the possibility of relevance to PSCI (Martin‐Ruiz et al. 2006); however, whether LTL is a causal determinant or primarily a proxy of systemic aging burden remains uncertain.

Inter‐individual variation in LTL is highly heritable and polygenic (Broer et al. 2013). Large genome‐wide association studies (GWAS) have identified multiple loci influencing LTL (Codd et al. 2021), enabling construction of polygenic risk scores (PRS) that provide a genetically anchored proxy of lifelong telomere maintenance capacity (Chatterjee et al. 2016; Choi et al. 2020; Choi and O'Reilly 2019). Importantly, East‐Asian populations remain under‐represented in telomere genetics, and stroke‐specific evaluations of LTL and LTL‐PRS in relation to PSCI are scarce (Dorajoo et al. 2019; Chang et al. 2021). We therefore investigated whether baseline LTL and LTL‐PRS are associated with 12‐month PSCI in a large, prospective Chinese cohort of patients with acute ischemic stroke (AIS) or transient ischemic attack (TIA). Given prior heterogeneity in literature and biological plausibility for context effects, we prespecified exploratory assessments of effect modification by age, sex, and stroke etiology (Liu et al. 2023; Demanelis et al. 2020; Gardner et al. 2014), recognizing that telomere‐outcome associations may vary across demographic strata and vascular phenotypes via mechanisms such as endothelial senescence and atherosclerosis (Rossiello et al. 2022).

## 2 Methods

### 2.1 Study Design and Participants

We analyzed data from the Third China National Stroke Registry (CNSR‐III), a nationwide, multicenter prospective registry that consecutively enrolled adults (≥18 years) hospitalized with AIS or TIA within 7 days of symptom onset in China between August 2015 and March 2018 (Wang et al. 2019). AIS and TIA were defined according to WHO criteria and confirmed by brain MRI or CT; silent cerebral infarction without symptoms or signs was excluded (Wang et al. 2019).

A prespecified CNSR‐III genetic substudy (the STROMICS genome study) collected peripheral blood samples during the index hospitalization, extracted leukocyte DNA, and generated deep whole‐genome sequencing (WGS) data (Cheng et al. 2023). Telomere metrics (LTL and LTL‐PRS) were derived from these WGS data as described below.

The impairment of cognition and sleep (ICONS) study is a prospective sub cohort nested within CNSR‐III, designed to characterize post‐stroke cognitive outcomes and sleep disorders through face‐to‐face assessments at prespecified follow‐up intervals (Wang et al. 2021). Between August 2015 and January 2018, ICONS screened CNSR‐III participants at 40 selected CNSR‐III sites and enrolled those meeting CNSR‐III eligibility criteria, with additional exclusions to ensure valid cognitive and sleep assessments (Wang et al. 2021). Specifically, ICONS excluded patients with a prior diagnosis of cognitive impairment, schizophrenia or psychotic disorders, illiteracy, or concomitant neurological or sensory conditions that could preclude reliable testing (e.g., severe aphasia defined as NIHSS item 9 > 2, severe unilateral neglect, visual impairment, hearing loss, dyslexia, or disorders of consciousness) (Wang et al. 2021). The ICONS eligibility criteria (including exclusion of patients with a documented prior diagnosis of cognitive impairment) and the prespecified follow‐up assessments have been used in prior publications from this cohort (Wang et al. 2021; Liao et al. 2022; Zuo et al. 2024; Li et al. 2021).

**FIGURE 1:**
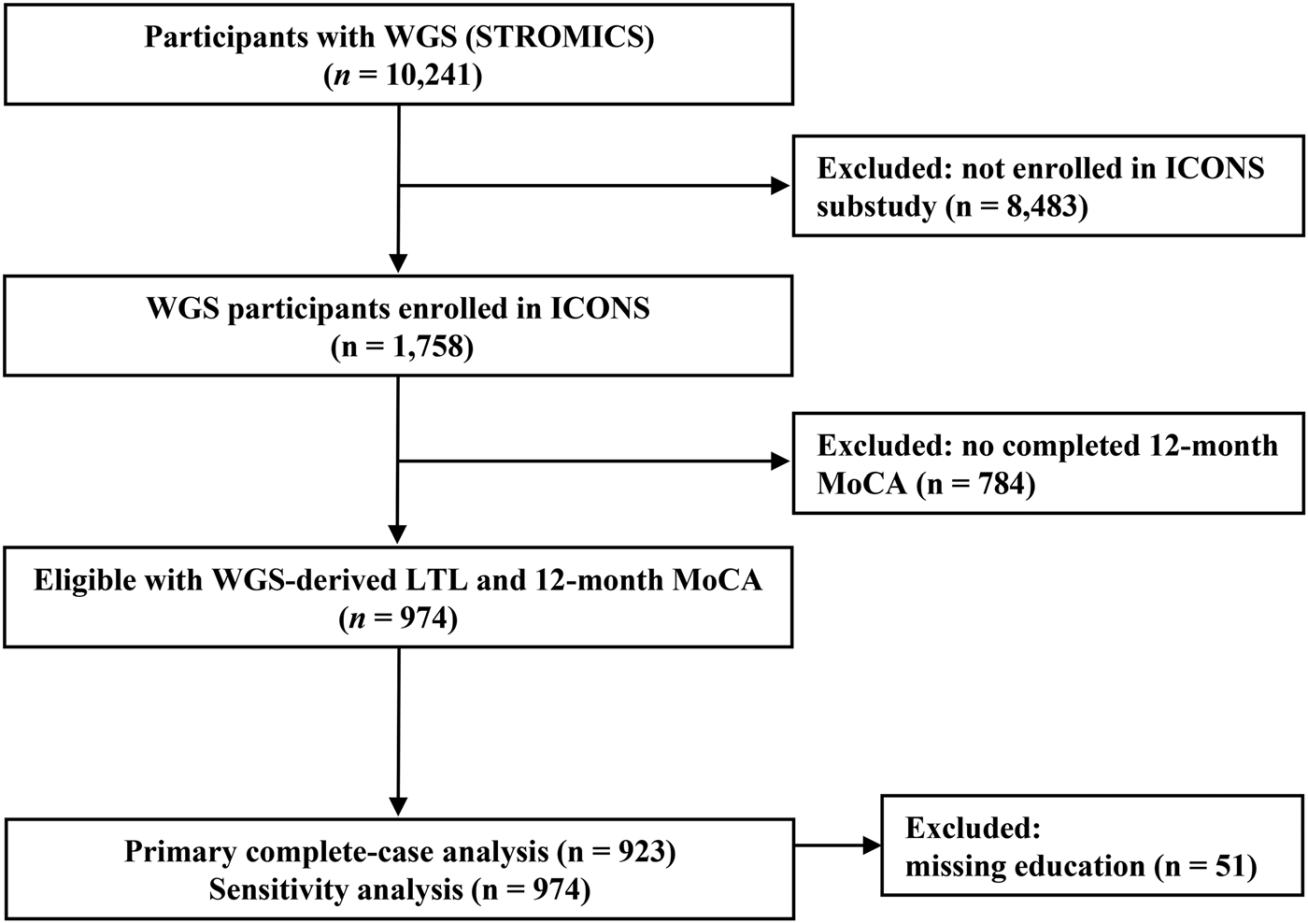
Flow diagram of participant selection. From the STROMICS whole‐genome sequencing (WGS) cohort, 1758 participants were enrolled in ICONS cognitive follow‐up; 974 had a 12‐month MoCA. For the primary norm‐referenced PSCI analysis, 51 participants with missing education were excluded (final *n* = 923); threshold‐based sensitivity analyses (MoCA ≤22 or ≤24) used *n* = 974.

For the present analysis, we included ICONS participants who were also enrolled in the CNSR‐III genetic substudy, had high‐quality WGS data available, and completed the face‐to‐face 12‐month cognitive assessment (Wang et al. 2021). The study flow diagram (Figure 1) details screening, exclusions, and derivation of the analysis sets, including the full cohort with WGS and 12‐month MoCA (*n* = 974) and the reduced cohort with complete demographic data required for the norm‐referenced PSCI definition (*n* = 923), which served as the primary analytic sample. The study was approved by the institutional review boards of all participating centers, and written informed consent was obtained from all participants or their legally authorized representatives.

### 2.2 Clinical Data and Assessments

Baseline data included demographics (age, sex, education level), vascular risk factors (hypertension, diabetes mellitus, dyslipidemia, atrial fibrillation, smoking status, alcohol consumption, body mass index), medical history (including prior stroke or TIA, coronary artery disease, heart failure), and medications at admission (antiplatelet agents, antihypertensives, lipid‐lowering agents, hypoglycemic drugs). Stroke severity on admission was assessed with the National Institutes of Health Stroke Scale (NIHSS). Index events were classified as IS or TIA, and IS subtypes were defined by Trial of Org 10172 in Acute stroke treatment (TOAST) criteria. All baseline and outcome assessments were conducted by personnel blinded to telomere and genetic data.

### 2.3 Cognitive Function Assessment

Participants’ cognitive status at 12 months post‐stroke was evaluated using the Montreal Cognitive Assessment (MoCA), a widely used cognitive screening tool in stroke populations (Nasreddine et al. 2005; Quinn et al. 2018). PSCI was defined a priori as a 12‐month MoCA total score below age‐, sex‐, and education‐specific normative cut‐offs derived from published large‐scale Chinese reference data (Supplementary Table S1) (An et al. 2018). This norm‐referenced definition corresponds to a demographically adjusted MoCA z score ≤ −1.5 and aligns with a recent post‐stroke study that operationalized PSCI using the same normative MoCA cut‐offs (Pan et al. 2023). Primary analyses were restricted to participants with available age, sex, and education information. For comparability and clinical interpretability, sensitivity analyses additionally applied fixed MoCA thresholds of ≤22 (Wei et al. 2023) and ≤24 (Liao et al. 2021). For these threshold‐based analyses, the standard MoCA education correction (+1 point for <12 years of education) was applied when education data were available (Nasreddine et al. 2005); participants with missing education were retained and analyzed using uncorrected MoCA scores. MoCA domain scores (attention, executive function, memory, language, and visuospatial function) were derived using standard item groupings (Lam et al. 2013). All cognitive evaluators were trained neurologists or neuropsychologists and were blinded to participants’ telomere length and genetic data.

### 2.4 Leukocyte Telomere Length Estimation

Peripheral blood was collected at baseline during the index hospitalization, typically within 24 h of admission, in participants enrolled within 7 days of symptom onset per CNSR‐III/ICONS criteria. Samples were processed locally and stored at −80°C before shipment for central sequencing. Deep WGS (30× coverage) was performed on leukocyte‐derived DNA at Beijing Tiantan Hospital as part of the prespecified CNSR‐III genetic sub study (STROMICS) (Cheng et al. 2023). LTL was quantified from these WGS data using TelSeq, which estimates mean telomere length from the proportion of telomeric reads with adjustment for sequencing depth and GC content (Ding et al. 2014). Raw sequencing data were processed using standardized CNSR‐III bioinformatics pipelines (Zhang et al. 2023), and sequencing quality control followed published STROMICS procedures (Cheng et al. 2023). TelSeq outputs LTL in kilobases (kb).

### 2.5 Polygenic Risk Score (LTL‐PRS) Construction

An LTL‐PRS was constructed using PRSice‐2 (Choi et al. 2020; Choi and O'Reilly 2019). Nineteen SNPs previously associated with LTL in Singaporean Chinese GWAS were initially selected (Dorajoo et al. 2019; Chang et al. 2021). Genotype data in the C3 cohort had undergone centralized quality control as part of the STROMICS genome study, with sample‐ and variant‐level thresholds (e.g., sample call rate ≥95%, variant call rate ≥99%, minor allele frequency ≥1%, and Hardy–Weinberg equilibrium *P* ≥ 1 × 10^−^^6^) (Cheng et al. 2023; Marees et al. 2018). Within our analytic dataset, allele‐frequency and strand/alignment checks were performed; one low‐frequency SNP failed and was excluded, leaving 18 SNPs for LTL‐PRS derivation (Supplementary Table S2). Linkage disequilibrium clumping used the 1000 Genomes East Asian reference panel (*r^2^* < 0.1 within 500 kb). SNPs were weighted by published GWAS‐derived β‐coefficients for LTL, and the resulting scores were standardized to z‐standardized (mean = 0, SD = 1). We evaluated LTL‐PRS performance in the parent CNSR‐III WGS cohort (*n* = 10,241) by regressing TelSeq‐estimated LTL on the standardized LTL‐PRS. The LTL‐PRS explained 0.36% of the variance in TelSeq‐estimated LTL (*R^2^* = 0.0036; *F* = 39.7; *p* < 0.0001), a proportion consistent with, albeit on the lower end of, the range typically reported for telomere PRS (Codd et al. 2021; Lewis and Vassos 2020).

### 2.6 Statistical Analyses

Baseline characteristics were summarized by LTL quartiles. Continuous variables were expressed as mean ± standard deviation (SD) if normally distributed or median (interquartile range, IQR) otherwise. Categorical variables were reported as counts (percentages). Across LTL quartiles, the Kruskal–Wallis test was used for continuous variables and the chi‐square test for categorical variables.

The primary outcome was PSCI at 12 months, defined using the demographically adjusted, norm‐referenced MoCA threshold. In sensitivity analyses, PSCI was alternatively defined using MoCA cut‐offs of ≤22 and ≤24. Associations with PSCI were examined using multivariable logistic regression for three LTL metrics: continuous LTL (per 1000 bp), quartile‐based LTL (Q1–Q4, defined by the cohort distribution: Q1 = 0–25%, Q2 = 25–50%, Q3 = 50–75%, Q4 = >75%; Q1 as the reference category), and standardized LTL‐PRS (per SD). Three nested models were fitted: an unadjusted model; a model adjusted for age, sex, and education; and a fully adjusted model additionally including admission NIHSS score, prior TIA, and TOAST subtype. Exploratory subgroup analyses were conducted by age (≤65 vs. >65 years), sex, and TOAST subtype using model‐based interaction terms. *P*‐values (including interaction *p*‐values) were considered nominal (two‐sided) and were not adjusted for multiple comparisons.

For secondary outcomes, each of the five MoCA subdomains was analyzed using ordinal (proportional‐odds) logistic regression, reporting common odds ratios (ORs) with 95% confidence intervals (CIs). The proportional‐odds assumption was evaluated using score tests (all *p* > 0.05). Analyses were conducted as complete‐case analyses using SAS version 9.4 (SAS Institute, Cary, NC, USA). Two‐sided *p*‐values < 0.05 were considered statistically significant for primary analyses.

## 3 Results

### 3.1 Baseline Characteristics

Among 10,241 participants in the CNSR‐III genetic sub study with WGS, 974 had baseline LTL estimated using TelSeq and 12‐month MoCA data available. A further 51 participants lacked education required for norm‐based classification and were therefore excluded from outcome modeling, yielding 923 individuals for the primary complete‐case analysis (Figure 1 **)**. Baseline characteristics of the descriptive cohort (*n* = 974) are summarized in Table 1. The mean age was 61.4 ± 11.1 years and 26.2% (*n* = 255) were women. Across LTL quartiles, there were significant differences in sex distribution (*p* = 0.008), history of TIA (*p* = 0.03), baseline NIHSS (*p* = 0.007), and TOAST subtypes (*p* = 0.03), with no differences in age, education, vascular risk factors, or lifestyle (all *p* > 0.05). Mean LTL was 2.24 kb (range, 1.77–2.75 kb) and showed a weak, non‐significant inverse correlation with age (Pearson *r* = −0.05; *p* = 0.09; Supplementary Figure S1). By construction, the standardized LTL‐PRS had a mean of 0 and a SD of 1.

**TABLE 1:** Baseline characteristics of the study cohort stratified by leukocyte telomere length quartiles (*n* = 974).

Characteristic	Total (*n* = 974)	Q1 (*n* = 228)	Q2 (*n* = 247)	Q3 (*n* = 243)	Q4 (*n* = 256)	*P*
Age (years), median (IQR)	61.0 (54.0, 69.0)	62.0 (53.5, 68.5)	63.0 (56.0, 71.0)	61.0 (53.0, 69.0)	60.0 (52.0, 68.0)	0.06
Women, *n* (%)	255 (26.2)	45 (19.7)	57 (23.1)	71 (29.2)	82 (32.0)	0.008
BMI (kg/m^2^), median (IQR)	24.8 (22.9, 26.8)	24.8 (22.6, 26.5)	25.0 (22.9, 26.9)	24.8 (23.4, 27.0)	24.8 (22.6, 27.0)	0.44
Current smoking, *n* (%)	352 (36.1)	96 (42.1)	87 (35.2)	85 (35.0)	84 (32.8)	0.17
Current drinking, *n*(%)	163 (16.7)	40 (17.5)	45 (18.2)	39 (16.1)	39 (15.2)	0.80
Level of education(y), *n*(%)	—	—	—	—	—	0.58
College or above	120 (12.3)	32 (14.0)	28 (11.3)	29 (11.9)	31 (12.1)	—
High school	228 (23.4)	52 (22.8)	59 (23.9)	54 (22.2)	63 (24.6)	—
Junior school	346 (35.5)	72 (31.6)	95 (38.5)	95 (30.1)	84 (32.8)	—
Primary school	229 (23.5)	55 (24.1)	51 (20.7)	54 (22.2)	69 (27.0)	—
Unknown	51 (5.2)	17 (7.5)	14 (5.7)	11 (4.5)	9 (3.5)	—
Systolic BP (mmHg), median (IQR)	147.5 (132.5, 161.0)	145.3 (131.5, 162.0)	147.5 (131.0, 160.0)	145.0 (132.0, 158.5)	149.3 (136.0, 165.3)	0.16
Diastolic BP (mmHg), median (IQR)	85.0 (78.0, 95.0)	85.0 (78.3, 95.0)	85.0 (77.5, 95.0)	85.0 (78.0, 93.5)	86.5 (79.0, 96.3)	0.63
NIHSS score on admission, median (IQR)	3 (1,5)	3 (2, 5)	3 (1, 5)	2 (1, 4)	3 (1, 4)	0.007
Pre‐stroke mRS score, median (IQR)	0 (0,1)	0 (0, 1)	0 (0, 1)	0 (0, 0)	0 (0, 1)	0.17
Medical history, *n* (%)	—	—	—	—	—	—
Stroke history	198 (20.3)	50 (21.9)	54 (21.9)	39 (16.1)	55 (21.5)	0.29
TIA history	35 (3.6)	5 (2.2)	16 (6.5)	9 (3.7)	5 (2.0)	0.03
Coronary heart disease	117 (12.0)	27 (11.8)	37 (15.0)	27 (11.1)	26 (10.2)	0.38
Heart failure	5 (3.7)	0 (0)	2 (5.1)	1 (2.9)	2 (6.3)	0.56
Atrial fibrillation	52 (5.3)	12 (5.3)	12 (4.9)	15 (6.2)	13 (5.1)	0.92
Hypertension	609 (62.5)	136 (59.7)	151 (61.1)	150 (61.7)	172 (67.2)	0.33
Diabetes	228 (23.4)	58 (25.4)	61 (24.7)	50 (20.6)	59 (23.1)	0.60
Dyslipidemia	101 (10.4)	31 (13.6)	24 (9.7)	23 (9.5)	23 (9.0)	0.33
Medication history, *n* (%)	—	—	—	—	—	—
Antiplatelet agents	196 (20.1)	55 (24.1)	48 (19.4)	51 (21.0)	42 (16.4)	0.20
Antihypertensive agents	443 (45.5)	92 (40.4)	115 (46.6)	109 (44.9)	127 (49.6)	0.23
Lipid‐lowering agents	128 (13.1)	37 (16.2)	31 (12.6)	27 (11.1)	33 (12.9)	0.41
Hypoglycemic drugs	184 (18.9)	45 (19.7)	47 (19.0)	39 (16.1)	53 (20.7)	0.59
Index event (*n*%)	—	—	—	—	—	0.06
AIS	877 (90.0)	214 (93.9)	222 (89.9)	210 (86.4)	231 (90.2)	—
TIA	97 (10.0)	14 (6.1)	25 (10.1)	33 (15.6)	25 (9.8)	—
TOAST classification, *n*(%)	—	—	—	—	—	0.03
Large‐artery atherosclerosis	246 (25.3)	63 (27.6)	74 (30.0)	58 (23.9)	51 (19.9)	—
Cardioembolism	62 (6.4)	9 (4.0)	19 (7.7)	21 (8.6)	13 (5.1)	—
Small‐vessel occlusion	232 (23.8)	49 (21.5)	45 (18.2)	62 (25.5)	76 (29.7)	—
Other determined etiology	8 (0.8)	2 (0.9)	2 (0.8)	0 (0)	4 (1.6)	—
Undetermined etiology	426 (43.7)	105 (46.1)	107 (43.3)	102 (42.0)	112 (43.8)	—

*Note*: Values are *n* (%) for categorical variables and median (IQR) for continuous variables. *P* values are from χ^2^ tests (categorical) and Kruskal–Wallis tests (continuous) comparing across LTL quartiles (Q1–Q4). *P* < 0.05 in bold. Fifty‐one participants lacked one or more covariates (age, sex, or education) required for norm‐based classification and were excluded from outcome modeling, yielding an analytic sample of *n* = 923 for outcome analyses.

Abbreviations: AIS, acute ischemic stroke; BMI, body mass index; BP, blood pressure; IQR, interquartile range; LTL, leukocyte telomere length;mRS, modified Rankin Scale; NIHSS, National Institutes of Health Stroke Scale;Q, quartile; TIA, transient ischemic attack; TOAST, Trial of ORG 10172 in Acute Stroke Treatment.

### 3.2 Association of LTL and LTL‐PRS With PSCI

**FIGURE 2:**
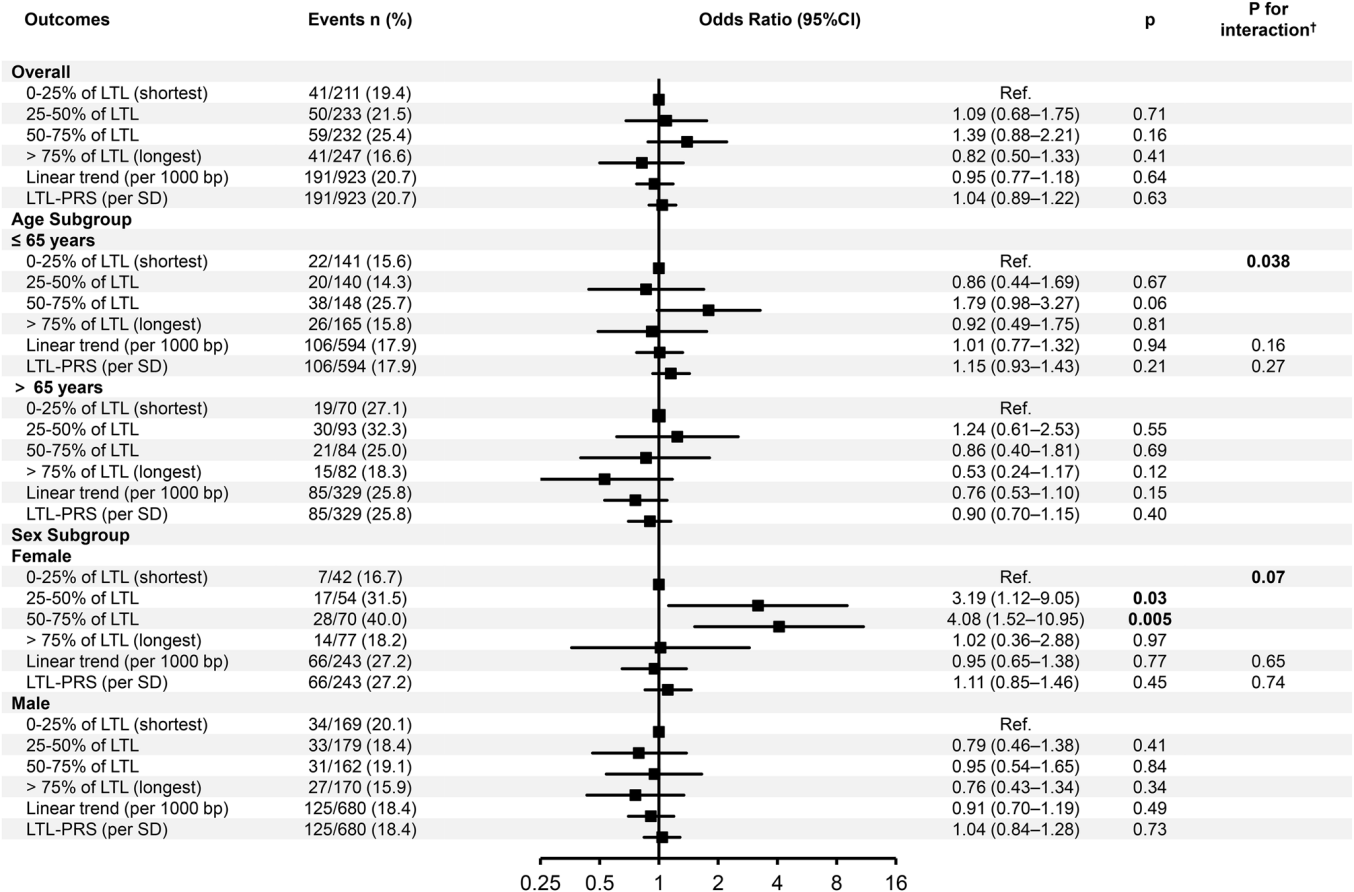
Associations of baseline leukocyte telomere length (LTL) and the LTL polygenic risk score (LTL‐PRS) with 12‐month post‐stroke cognitive impairment (PSCI), overall and stratified by age and sex. Odds ratios (ORs) and 95% confidence intervals (CIs) are from fully adjusted logistic regression. LTL is modelled as quartiles (Q2–Q4 vs. Q1), per 1000 bp increase (linear trend), and LTL‐PRS per SD. Stratified models omit the stratification variable. Models were adjusted for age, sex, education, prior transient ischemic attack (TIA), admission NIH stroke scale (NIHSS) score, and TOAST subtype. †*P* for interaction was from a Wald test of the exposure × subgroup term in the fully adjusted model and is nominal (not adjusted for multiple testing). Ref., reference category.

At 12‐month follow‐up, PSCI occurred in 191 of 923 participants (20.7%; Figure 2, Supplementary Table S3). In fully adjusted models (adjusted for age, sex, education, prior TIA, baseline NIHSS, TOAST), LTL was not associated with PSCI whether analyzed continuously or by quartiles (per 1000 bp increase: OR = 0.95; 95% CI, 0.77–1.18; *p* = 0.64; all quartile comparisons vs. Q1: *p* > 0.20). Likewise, LTL‐PRS showed no overall association with PSCI (per 1 SD: OR = 1.04; 95% CI, 0.89–1.22; *p* = 0.63).

### 3.3 Exploratory Subgroup Analyses

Exploratory subgroup analyses suggested potential heterogeneity; however, these findings should be interpreted cautiously because subgroup comparisons and interaction tests were not adjusted for multiple testing and are therefore hypothesis‐generating.

In age‐stratified analyses, an age × LTL quartile interaction was observed in the fully adjusted model (*p* for interaction = 0.038; Wald test). Among patients ≤65 years, the third LTL quartile showed a trend toward higher odds of PSCI relative to Q1 (OR 1.79; 95% CI, 0.98–3.27; *p* = 0.06), whereas among those >65 years the fourth quartile showed a trend toward lower odds (OR 0.53; 95% CI, 0.24–1.17; *p* = 0.12; Figure 2; Supplementary Table S4).

In sex‐stratified models, women in the third LTL quartile had higher odds of PSCI than those in Q1 (OR 4.08; 95% CI, 1.52–10.95; *p* = 0.005), whereas no quartile associations were observed in men (*p* > 0.50). The LTL × sex interaction did not reach conventional significance (*p* for interaction = 0.07; fully adjusted model; Figure 2; Supplementary Table S5).

**FIGURE 3:**
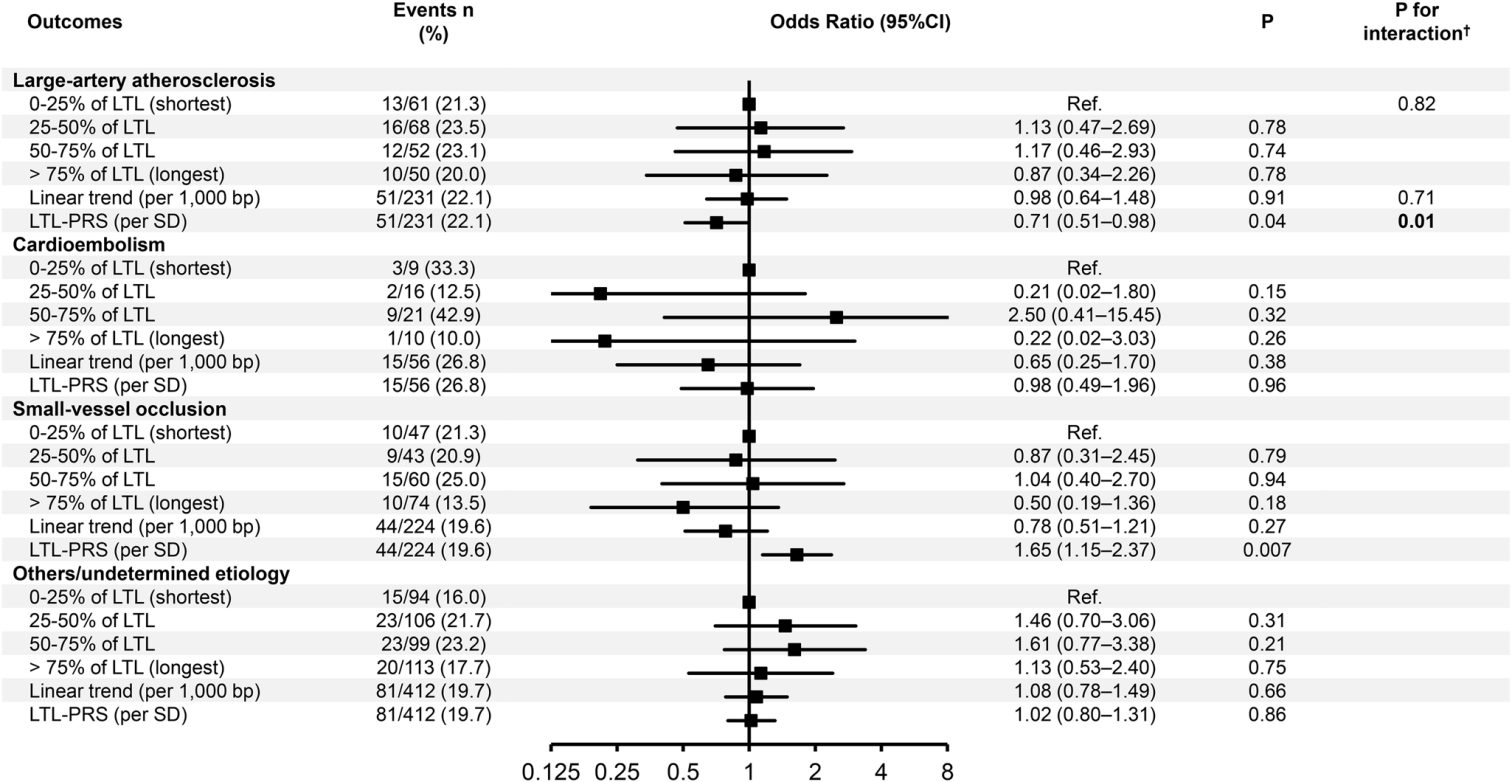
Associations of baseline leukocyte telomere length (LTL) and the LTL polygenic risk score (LTL‐PRS) with 12‐month post‐stroke cognitive impairment (PSCI) by TOAST subtype. Odds ratios (ORs) and 95% confidence intervals (CIs) are from fully adjusted logistic regression; subtype‐specific models omit TOAST as a covariate. Estimates are shown for LTL quartiles (Q2–Q4 vs. Q1), per 1000 bp increase (linear trend), and LTL‐PRS per SD. Models were adjusted for age, sex, education, prior transient ischemic attack (TIA), and admission NIH stroke scale (NIHSS) score. †P for interaction was from a Wald test of the exposure × subtype term in the fully adjusted model and is nominal (not adjusted for multiple testing). Ref., reference category.

Stroke etiology modified the association between LTL‐PRS and PSCI (*p* for interaction = 0.01; fully adjusted model). Higher LTL‐PRS was associated with lower odds of PSCI in large‐artery atherosclerosis (OR = 0.71; 95% CI, 0.51–0.98; *p* = 0.04) but higher odds in small‐vessel occlusion (OR = 1.65; 95% CI, 1.15–2.37; *p* = 0.007), with no clear associations in other TOAST subtypes (Figure 3; Supplementary Table S6). No subtype‐specific interactions were observed for baseline LTL.

### 3.4 Cognitive Subdomain Outcomes

Associations between telomere metrics and individual MoCA subdomains were weak and highly sensitive to covariate adjustments (Table 2; Supplementary Table S7). For executive function, the second LTL quartile showed borderline poorer performance in the model adjusted for age, sex, and education (OR = 1.43; 95% CI, 0.99–2.07; *p* = 0.05), which attenuated after full adjustment (OR = 1.40; 95% CI, 0.96–2.03; *p* = 0.06). A similar borderline trend was observed for the LTL‐PRS in fully adjusted models (OR = 1.13; 95% CI, 0.997–1.29; *p* = 0.06). No meaningful associations emerged for attention, memory, language, or visuospatial subscores (all *p* > 0.05).

**TABLE 2:** Associations of leukocyte telomere length (LTL) and the LTL polygenic risk score (LTL‐PRS) with MoCA domain scores at 12 months.

Outcomes	Model 1: cOR (95% CI)	*P*	Model 2: cOR (95% CI)	*P*
Attention	—	—	—	—
0–25% of LTL (shortest)	Ref.	—	Ref.	—
25–50% of LTL	1.02 (0.70–1.49)	0.74	1.07 (0.73–1.57)	0.62
50–75% of LTL	0.99 (0.67–1.45)	0.96	1.03 (0.70–1.53)	0.84
> 75% of LTL (longest)	0.92 (0.62–1.35)	0.59	0.94 (0.64–1.39)	0.54
Linear trend (per 1000 bp)	0.96 (0.81–1.15)	0.68	0.97 (0.82–1.16)	0.76
LTL‐PRS (per SD)	1.04 (0.91–1.19)	0.57	1.03 (0.90–1.17)	0.72
Executive	—	—	—	—
0%–25% of LTL (shortest)	Ref.	—	Ref.	—
25–50% of LTL	1.43 (0.99–2.07)	0.05	1.40 (0.96–2.03)	0.06
>50–75% of LTL	1.22 (0.84–1.77)	0.54	1.21 (0.83–1.76)	0.54
> 75% of LTL (longest)	0.96 (0.66–1.40)	0.15	0.96 (0.65–1.40)	0.16
Linear trend (per 1000 bp)	0.97 (0.82–1.16)	0.76	0.97 (0.82–1.15)	0.74
LTL‐PRS (per SD)	1.14 (1.01–1.30)	0.04	1.13 (0.997–1.29)	0.06
Language	—	—	—	—
0–25% of LTL (shortest)	Ref.	—	Ref.	—
25–50% of LTL	1.11 (0.79–1.56)	0.58	1.09 (0.77–1.54)	0.74
50–75% of LTL	1.25 (0.89–1.77)	0.08	1.27 (0.90–1.79)	0.08
> 75% of LTL (longest)	0.87 (0.61–1.22)	0.07	0.89 (0.63–1.26)	0.11
Linear trend (per 1000 bp)	0.91 (0.78–1.07)	0.25	0.92 (0.79–1.08)	0.32
LTL‐PRS (per SD)	1.13 (1.00–1.27)	0.047	1.12 (0.99–1.26)	0.07
Memory	—	—	—	—
0–25% of LTL (shortest)	Ref.	—	Ref.	—
25–50% of LTL	1.34 (0.97–1.84)	0.11	1.35 (0.98–1.87)	0.13
50–75% of LTL	1.26 (0.92–1.75)	0.30	1.31 (0.95–1.81)	0.23
> 75% (longest) of LTL	1.01 (0.73–1.38)	0.19	1.03 (0.75–1.42)	0.23
Linear trend (per 1000 bp)	1.00 (0.87–1.16)	0.97	1.02 (0.88–1.18)	0.82
LTL‐PRS (per SD)	1.02 (0.91–1.14)	0.77	1.01 (0.90–1.13)	0.86
Visuospatial	—	—	—	—
0–25% of LTL (shortest)	Ref.	—	Ref.	—
25–50% of LTL	1.25 (0.86–1.83)	0.26	1.29 (0.88–1.89)	0.29
50–75% of LTL	1.32 (0.90–1.93)	0.11	1.39 (0.95–2.03)	0.08
> 75% of LTL (longest)	0.89 (0.60–1.32)	0.07	0.94 (0.63–1.39)	0.11
Linear trend (per 1000 bp)	0.97 (0.81–1.15)	0.69	0.99 (0.83–1.18)	0.92
LTL‐PRS (per SD)	1.14 (0.996–1.29)	0.06	1.12 (0.985–1.28)	0.08

*Note*: Estimates derive from proportional‐odds logistic regression for each MoCA subdomain; the proportional‐odds assumption held for all subdomains (score‐test *p* > 0.05). Model 1: adjusted for age, sex, and education. Model 2: Model 1 plus history of TIA, baseline NIHSS score, and TOAST subtype. LTL‐PRS is z‐standardized; effects are per SD. Linear trend for LTL is per 1000 bp. Bold indicates *p* < 0.05.

Abbreviations: cOR, common odds ratio; CI, confidence interval; LTL, leukocyte telomere length; LTL‐PRS, leukocyte telomere length polygenic risk score; MoCA, Montreal Cognitive Assessment; Q, quartile; Ref., reference; SD, standard deviation; TIA, transient ischemic attack; NIHSS, National Institutes of Health Stroke Scale; TOAST, Trial of ORG 10172 in Acute Stroke Treatment.

### 3.5 Sensitivity Analyses

Results were materially unchanged when PSCI was alternatively defined using fixed MoCA cut‐offs of ≤22 and ≤24 (Supplementary Tables S8–S9).

## 4 Discussion

In this multicenter cohort, neither baseline LTL nor a limited‐variant LTL‐PRS independently predicted 12‐month PSCI after multivariable adjustment. These null overall effects are consistent with the predominantly small and heterogeneous associations between LTL and cognitive outcomes reported in population cohorts and meta‐analyses (Hägg et al. 2017; Gampawar et al. 2022, Liu et al. 2023). Importantly, the null LTL‐PRS finding should be interpreted in light of the PRS's limited ability to proxy genetically determined LTL in this cohort (*R^2^* = 0.0036; ∼0.36% for TelSeq‐estimated LTL), which would be expected to attenuate any downstream PRS–outcome association and reduce power to detect modest effects. Accordingly, our data primarily argue against the clinical prognostic utility of the current limited‐variant LTL‐PRS for PSCI, rather than against a potential biological contribution of telomere‐related genetics per se. Overall, these findings do not support single‐time‐point peripheral LTL or a sparse LTL‐PRS as stand‐alone prognostic tools for PSCI and instead favor integrated models that combine aging biology with vascular, inflammatory, and imaging markers to improve risk stratification (El Husseini et al. 2023; Filler et al. 2024). Methodological differences likely contribute to discrepant findings across studies, including assay modality (PCR versus WGS‐derived estimates such as TelSeq), sample size and setting (single‐center versus registry‐based), and the timing of blood sampling relative to acute illness.

Prior literature on telomeres and cognition has yielded mixed results: several studies link shorter telomeres to faster cognitive decline and higher dementia risk (Hägg et al. 2017; Martin‐Ruiz et al. 2006), whereas others suggest more complex, potentially U‐shaped relationship (Roberts et al. 2014; Fani et al. 2020). In exploratory analyses stratified by age, the LTL–PSCI association appeared to differ across age groups (*p* for interaction = 0.038). Among patients ≤65 years, Q3 showed higher odds of PSCI relative to Q1, whereas among those >65 years, Q4 showed a non‐significant trend toward lower odds. These stratum‐specific estimates were imprecise and should be interpreted as hypothesis‐generating. This pattern is compatible with prior work suggesting that telomere–cognition associations may be more detectable in midlife than in older age, when multiple aging pathways converge (Hägg et al. 2017; Gampawar et al. 2022; Liu et al. 2023).

In exploratory analyses stratified by sex, women in Q3 had higher odds of PSCI than those in Q1, whereas no clear quartile pattern was observed in men; the sex × LTL interaction was borderline (*p* for interaction = 0.07). Given known sex differences in telomere biology and immune aging, this signal is biologically plausible but requires replication in adequately powered, prespecified analyses (Taheri et al. 2022).

In exploratory analyses by TOAST subtype, the association between LTL‐PRS and PSCI differed across etiologies (*p* for interaction = 0.01), with a protective direction in large‐artery atherosclerosis and an adverse direction in small‐vessel occlusion. Because these subtype analyses were exploratory and not adjusted for multiple testing, the findings should be interpreted cautiously and considered hypothesis‐generating pending replication. Etiology‐specific effects are biologically conceivable given links between telomere biology, endothelial senescence, and vascular dysfunction (Rossiello et al. 2022; Minamino et al. 2002), but large‐scale genetic studies primarily implicate canonical telomere maintenance pathways; therefore, independent validation is required before drawing subtype‐specific inferences (Codd et al. 2021).

Mechanistically, telomere shortening activates DNA‐damage response (e.g., ATM‐p53‐p21), driving cellular senescence and a pro‐inflammatory senescent‐associated secretory phenotype (SASP) that contributes to chronic low‐grade “inflammaging” (Herbig et al. 2004; Coppé et al. 2010; Franceschi et al. 2018). In the brain, chronic neuroinflammation and neurovascular‐unit dysfunction promote neurodegeneration and impede repair (Heneka et al. 2015; Iadecola 2017). Superimposed upon this background, ASI induces intense oxidative stress, blood‐brain barrier disruption and systemic immune activation (Iadecola 2017; Chamorro et al. 2012). Such processes may obscure modest telomere‐mediated effects during early recovery, potentially explaining null main effects despite biologically plausible subgroup signals.

At the domain level, associations between telomere metrics and executive function were weak and attenuated after full adjustment. This echoes reports linking shorter telomeres to executive and processing‐speed vulnerability (Hägg et al. 2017; Yu et al. 2020), yet likely reflects the limited granularity and ceiling effects of MoCA composites, which collapse trail‐making, phonemic fluency and abstraction into a single composite [31, 36]. Future work should employ comprehensive neuropsychological batteries alongside multimodal imaging to test whether telomere metrics track fronto‐subcortical integrity and network efficiency.

An additional observation was that baseline LTL was not inversely correlated with age in this cohort. Similar attenuation has been reported in clinical samples (Martin‐Ruiz et al. 2006), and may reflect survivorship bias or stroke‐related shifts in leukocyte composition that obscure typical age‐telomere gradients. Longitudinal sampling across broader age ranges will be required to disentangle illness effects from intrinsic telomere dynamics.

This study has notable strengths, including a prospective nationwide multicenter stroke cohort, rigorous PSCI ascertainment using age‐, sex‐, and education‐adjusted norms with domain‐level analyses, parallel phenotypic (LTL) and genetic (LTL‐PRS) assessments, standardized procedures with outcome assessors blinded to telomere and genetic data, and multivariable models adjusting for demographic, vascular, and clinical confounders. Several limitations merit emphasis. Methodological constraints include: (1) LTL was estimated once in peripheral blood, and cross‐tissue correlations are modest; longitudinal change may be more informative than absolute length; (2) the 18‐SNP LTL‐PRS explained only ∼0.36% of the variance in TelSeq‐estimated LTL, indicating minimal predictive capacity for individual‐level telomere length and likely attenuation of any downstream PRS–outcome association; thus, the null overall LTL‐PRS finding should not be over‐interpreted as biological irrelevance but instead highlights the need for more predictive genome‐wide scores and larger samples to resolve modest genetic contributions with adequate precision; (3) LTL estimates were derived from leukocyte DNA collected during the index hospitalization, when acute inflammation and shifts in circulating leukocyte subpopulations may increase variability in leukocyte‐derived LTL estimates and bias associations toward the null; and (4) attrition bias cannot be excluded, as participants who died or were lost to follow‐up may have differed systematically from those completing the 12‐month cognitive assessment. Interpretative limitations include: (5) PSCI was operationalized using a MoCA‐based, norm‐referenced threshold, which captures a screening construct rather than a clinical diagnosis; and (6) interaction and subgroup analyses were exploratory and not adjusted for multiple comparisons, increasing the risk of false‐positive findings; subgroup signals should therefore be regarded as hypothesis‐generating and require confirmation in independent, adequately powered, preregistered cohorts.

## 5 Conclusions

In this multicenter cohort, neither baseline leukocyte LTL nor an 18‐variant LTL‐PRS independently predicted 12‐month PSCI. Exploratory heterogeneity by age, sex, and stroke subtype was hypothesis‐generating and requires replication in larger, preregistered cohorts. Overall, these results do not support single time‐point telomere metrics as stand‐alone prognostic markers for PSCI. Future work should prioritize integrated, longitudinal, multi‐omic models, potentially incorporating telomere dynamics alongside genomic, proteomic, and imaging measures, to improve individualized risk prediction and clarify mechanisms.

## Author Contributions

**Kunying Zhao**: conceptualization, investigation, methodology, formal analysis, visualization, writing – original draft, writing – review and editing. **Yanfeng Shi**: conceptualization, methodology, software, formal analysis, writing – review and editing. **Hongyi Yan**: formal analysis, software, methodology, visualization, writing – review and editing. **Hongyu Zhou**: conceptualization, investigation. **Jiejie Li**: writing – review and editing. **Si Cheng**: methodology. **Zhe Xu**: methodology. **Yuesong Pan**: methodology, supervision. **Zixiao Li**: project administration, supervision. **Xia Meng**: funding acquisition. **Hao Li**: resources. **Xiaoling Liao**: resources. **Jing Jing**: writing – review and editing, project administration, supervision. **Yongjun Wang**: conceptualization, writing – review and editing, project administration, supervision, resources, funding acquisition.

## Funding

This study is supported by the National Key R&D Program of China (2022YFC2502400, 2022YFC2502404), the National Natural Science Foundation of China (82111530203), Chinese Academy of Medical Sciences Innovation Fund for Medical Sciences (2019‐I2M‐5‐029).

## Ethics Statement

Approved by the Ethics Committee of Beijing Tiantan Hospital (No. KY2015‐001‐01). The study adhered to the Declaration of Helsinki.

## Consent

Written informed consent was obtained from all participants or their legally authorised representatives.

## Conflicts of Interest

The authors declare no conflicts of interest.

## Supporting information




**Supplementary Material**: brb371435‐sup‐0001‐SuppMat.docx

## Data Availability

De‐identified data, analysis code and materials that support the findings of this study are available from the corresponding author on reasonable request for research purposes, subject to institutional and ethical approvals.
