# Supplementary material for: Leukocyte Telomere Length and Its Polygenic Risk Score in Post‐Stroke Cognitive Impairment: Evidence From a Multicenter Cohort Study
Source: Brain Behav. 2026 May 14;16(5):e71435. doi: 10.1002/brb3.71435 (PMC13175197; doi:10.1002/brb3.71435)
Supplement: Supplementary file 1 — Supplementary Material: brb371435‐sup‐0001‐SuppMat.docx [file BRB3-16-e71435-s001.docx]

**Online Supplementary Material**

**Leukocyte Telomere Length and Its Polygenic Risk Score in Post-Stroke Cognitive Impairment: Evidence from a Multicenter Cohort Study**

Kunying Zhao ^a, c, 1^, Yanfeng Shi ^b, 1^, Hongyi Yan ^b, 1^, Hongyu Zhou ^a^, Jiejie Li ^a, b^, Si Cheng ^a, b^, Zhe Xu ^b^, Yuesong Pan ^b^, Zixiao Li ^a, b^, Xia Meng ^a, b^, Hao Li ^b^, Xiaoling Liao ^a, b^, Jing Jing ^a, b,^ *,Yongjun Wang ^a, b,^ *

**Affiliation:**

^a^ Department of Neurology, Beijing Tiantan Hospital, Capital Medical University, Beijing, China;

^b^ China National Clinical Research Center for Neurological Diseases, Beijing, China;

^c^ Department of Neurology, Beijing Fengtai Hospital, Beijing, China.

**Corresponding author:**

Yongjun Wang, Professor, MD

Address: Department of Neurology, Beijing Tiantan Hospital, Capital Medical University, No.119 South 4th Ring West Road, Fengtai District, Beijing, 100070, China

Email: [yongjunwang@ncrcnd.org.cn](mailto:yongjunwang@ncrcnd.org.cn)

Jing Jing, Professor, MD

Address: Department of Neurology, Beijing Tiantan Hospital, Capital Medical University, No.119 South 4th Ring West Road, Fengtai District, Beijing, 100070, China

Email: [jingj_bjttyy@163.com](mailto:jingj_bjttyy@163.com)

^1^ These authors contributed equally to this work.

**Abbreviations**

WGS, whole-genome sequencing;

LTL, leukocyte telomere length;

PRS, polygenic risk score;

LTL-PRS, leukocyte telomere-length polygenic risk score;

MoCA, Montreal Cognitive Assessment;

PSCI, post-stroke cognitive impairment;

NIHSS, National Institutes of Health Stroke Scale;

TIA, transient ischaemic attack;

TOAST, Trial of ORG 10172 in Acute Stroke Treatment;

LAA, large-artery atherosclerosis;

SVO, small-vessel occlusion;

OR, odds ratio;

cOR, common odds ratio;

CI, confidence interval;

SD, standard deviation;

kb, kilobase;

GWAS, genome-wide association study;

SNP, single nucleotide polymorphism.

**Supplementary Table S1.** Demographically stratified MoCA reference cut-offs derived from published Chinese normative data, used to define PSCI in the primary analysis.

**Supplementary Table S2.** SNPs Included in the Telomere Length Polygenic Risk Score (TL-PRS) for the Singaporean Chinese Cohort (hg19)

**Supplementary Figure S1.** Distribution of leukocyte telomere length with chronological aging.

**Supplementary Table S3.** Association of leukocyte telomere length (LTL) and LTL polygenic risk score with post-stroke cognitive impairment

**Supplementary Table S4.** Association of leukocyte telomere length (LTL) and LTL polygenic risk score with post-stroke cognitive impairment stratified by age group (≤ 65 vs > 65 years)

**Supplementary Table S5.** Association of leukocyte telomere length (LTL) and LTL polygenic risk score with post-stroke cognitive impairment stratified by sex

**Supplementary Table S6.** Association of leukocyte telomere length (LTL) and LTL polygenic risk score with post-stroke cognitive impairment stratified by TOAST subtype

**Supplementary Table S7.** Association between leukocyte telomere length and MoCA sub-domain scores (ordinal logistic regression, unadjusted model)

**Supplementary Table S8.** Sensitivity analysis using an alternative PSCI definition (MoCA ≤22)

**Supplementary Table S9.** Sensitivity analysis using an alternative PSCI definition (MoCA ≤24)

**Supplementary Table S1. Demographically stratified MoCA reference cut-offs derived from published Chinese normative data, used to define PSCI in the primary analysis.**

| **Age** | **Primary school and below** | | **Junior school** | | **High school** | | **College and above** | |
| --- | --- | --- | --- | --- | --- | --- | --- | --- |
|  | Male | Female | Male | Female | Male | Female | Male | Female |
| 50-54 | 18 | 18 | 20 | 20 | 21 | 21 | 22 | 23 |
| 55-59 | 18 | 17 | 20 | 19 | 21 | 21 | 22 | 22 |
| 60-64 | 18 | 17 | 20 | 19 | 21 | 20 | 22 | 22 |
| 65-70 | 18 | 17 | 20 | 19 | 21 | 20 | 22 | 22 |

**Source:** Cut-offs were extracted from published regression-based normative dataset, in which age-, sex-, and education-specific reference values were defined as 1.5 × RMSE below the demographically predicted mean [32]. This normative framework has been applied in post-stroke cohorts (e.g., [33]).

**Supplementary Table S2. SNPs Included in the Leukocyte Telomere Length Polygenic Risk Score (LTL-PRS) for the Singaporean Chinese Cohort (hg19) [1,2]**

| **SNP** | **CHROM** | **POS** | **REF** | **ALT** | **AF** | **GWAS β** | **Included in PRS** |
| --- | --- | --- | --- | --- | --- | --- | --- |
| rs3219104 | chr1 | 226374920 | A | C | 0.568 | 0.074 | Yes |
| rs11890390 | chr2 | 54258545 | C | T | 0.2 | 0.048 | Yes |
| rs2293607 | chr3 | 169764547 | T | C | 0.554 | 0.12 | Yes |
| rs10857352 | chr4 | 163180330 | A | G | 0.737 | 0.064 | Yes |
| rs7705526 | chr5 | 1285859 | C | A | 0.419 | 0.118 | Yes |
| rs79314063 | chr7 | 124841114 | C | G | 0.011 | 0.32 | Yes |
| rs7776744 | chr7 | 124959695 | A | G | 0.602 | 0.065 | Yes |
| rs28365964 | chr8 | 73008648 | T | C | 0.017 | 0.27 | Yes |
| rs79617270 | chr8 | 73065909 | A | G | 0.01 | 0.376 | Yes |
| rs7095953 | chr10 | 99514668 | T | C | 0.406 | 0.047 | Yes |
| rs139620151 | chr10 | 103833670 | G | A | 0.002 | 0.394 | **No** |
| rs12415148 | chr10 | 103920828 | T | C | 0.072 | 0.204 | Yes |
| rs41293836 | chr14 | 24252121 | C | T | 0.094 | 0.233 | Yes |
| rs2302588 | chr14 | 72938044 | G | C | 0.198 | 0.053 | Yes |
| rs2967374 | chr16 | 82176256 | A | G | 0.822 | 0.056 | Yes |
| rs1001761 | chr18 | 662103 | G | A | 0.672 | 0.034 | Yes |
| rs7253490 | chr19 | 22110904 | C | A | 0.279 | 0.043 | Yes |
| rs41309367 | chr20 | 63678201 | C | T | 0.255 | 0.058 | Yes |

**Note**: This table lists the SNPs used to construct the LTL-PRS based on GWAS of leukocyte telomere length in a Singaporean Chinese population. Columns are as follows: SNP, dbSNP identifier (rsID); Chr, chromosome; Pos, base-pair position (hg19); Ref, reference allele; Alt, alternative (effect) allele; AF, alternative allele frequency.

**References:** 1. Dorajoo R, Chang X, Gurung RL, et al. Loci for human leukocyte telomere length in the Singaporean Chinese population and trans-ethnic genetic studies. *Nat Commun* 2019; **10**: 2491.

2. Chang X, Gurung RL, Wang L, et al. Low frequency variants associated with leukocyte telomere length in the Singapore Chinese population. *Commun Biol* 2021; **4**: 519.


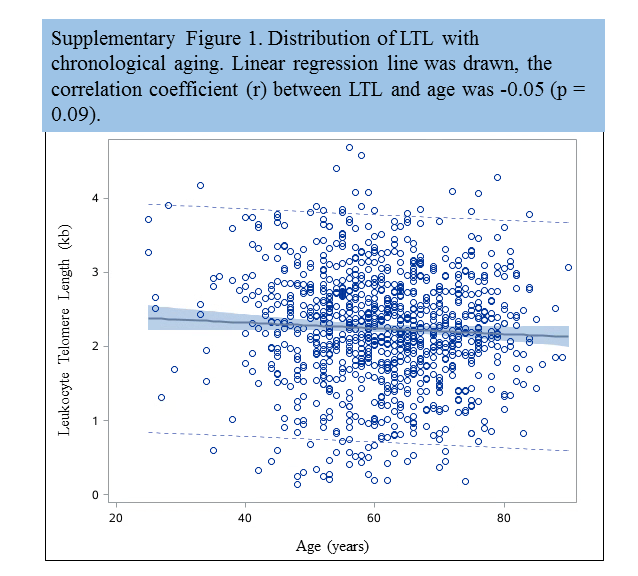


**Supplementary Table S3. Association of leukocyte telomere length (LTL) and LTL polygenic risk score with post-stroke cognitive impairment (overall cohort; *n* = 923)**

| **Exposure** | **Events n (%)** | **Model 1**  **OR (95% CI)** | ***P*** | **Model 2**  **OR (95% CI)** | ***P*** |
| --- | --- | --- | --- | --- | --- |
| 0-25% of LTL (shortest) | 41/211 (19.4) | Ref. |  | Ref. |  |
| > 25-50% of LTL | 50/233 (21.5) | 1.13 (0.71–1.80) | 0.60 | 1.06 (0.67–1.70) | 0.79 |
| > 50-75% of LTL | 59/232 (25.4) | 1.41 (0.90–2.22) | 0.13 | 1.33 (0.84–2.11) | 0.22 |
| > 75% of LTL (longest) | 41/247 (16.6) | 0.83 (0.51–1.33) | 0.43 | 0.78 (0.48–1.27) | 0.33 |
| Linear trend (per 1,000 bp) | 191/923 (20.7) | 0.95 (0.78–1.17) | 0.64 | 0.93 (0.76–1.15) | 0.52 |
| LTL-PRS (per SD) | 191/923 (20.7) | 1.04 (0.89–1.22) | 0.61 | 1.05 (0.90–1.23) | 0.54 |

**Note:** Model 1 = unadjusted; Model 2 = adjusted for age, sex, and education. LTL-PRS was z-standardised within the analytic cohort and modelled per 1 SD increase. The linear trend for LTL was modelled per 1,000 bp. Two-sided p values are reported.

**Supplementary Table S4. Association of leukocyte telomere length (LTL) and LTL** **polygenic risk score with post-stroke cognitive impairment stratified by age group (≤ 65 vs > 65 years)**

| **Exposure** | **Events n (%)** | **Model 1**  **OR (95% CI)** | ***P*** | **Model 2**  **OR (95% CI)** | ***P*** |
| --- | --- | --- | --- | --- | --- |
| **≤ 65 years** |  |  |  |  |  |
| 0-25% (shortest) | 22/141 (15.6) | Ref. |  | Ref. |  |
| 25-50% | 20/140 (14.3) | 0.90 (0.47–1.74) | 0.76 | 0.84 (0.43–1.63) | 0.60 |
| 50-75% | 38/148 (25.7) | **1.87 (1.04–3.36)** | **0.04** | 1.70 (0.94–3.09) | 0.08 |
| > 75% (longest) | 26/165 (15.8) | 1.01 (0.55–1.88) | 0.97 | 0.86 (0.46–1.62) | 0.64 |
| Linear trend (per 1,000 bp) | 106/594 (17.9) | 1.04 (0.81–1.35) | 0.74 | 0.98 (0.75–1.28) | 0.87 |
| LTL-PRS (per SD) | 106/594 (17.9) | 1.12 (0.90–1.39) | 0.31 | 1.17 (0.94–1.45) | 0.16 |
| **> 65 years** |  |  |  |  |  |
| 0-25% of LTL (shortest) | 19/70 (27.1) | Ref. |  | Ref. |  |
| 25-50% of LTL | 30/93 (32.3) | 1.28 (0.65–2.53) | 0.48 | 1.20 (0.60–2.42) | 0.61 |
| 50-75% of LTL | 21/84 (25.0) | 0.90 (0.44–1.84) | 0.76 | 0.82 (0.39–1.72) | 0.60 |
| > 75% of LTL (longest) | 15/82 (18.3) | 0.60 (0.28–1.30) | 0.19 | 0.51 (0.23–1.13) | 0.10 |
| Linear trend (per 1,000 bp) | 85/329 (25.8) | 0.81 (0.57–1.15) | 0.24 | 0.75 (0.52–1.09) | 0.13 |
| LTL-PRS (per SD) | 85/329 (25.8) | 0.96 (0.76–1.22) | 0.76 | 0.91 (0.71–1.17) | 0.48 |

**Note:** Model 1 = unadjusted; Model 2 = adjusted for age, sex, and education. LTL-PRS is z-standardised within the analytic cohort; effects are per 1 SD. Linear trend for LTL is per 1,000 bp. Two-sided *p*-values are nominal (not adjusted for multiple testing).

**Supplementary Table S5. Association of leukocyte telomere length (LTL) and LTL polygenic risk score with post-stroke cognitive impairment stratified by sex**

| **Exposure** | **Events n (%)** | **Model 1**  **OR (95% CI)** | ***P*** | **Model 2**  **OR (95% CI)** | ***P*** |
| --- | --- | --- | --- | --- | --- |
| **Female** |  |  |  |  |  |
| 0-25% of LTL (shortest) | 7/42 (16.7) | Ref. |  | Ref. |  |
| 25-50% of LTL | 17/54 (31.5) | 2.30 (0.85–6.21) | 0.10 | 2.50 (0.91–6.90) | 0.07 |
| 50-75% of LTL | 28/70 (40.0) | **3.33 (1.30–8.55)** | **0.01** | **3.42 (1.31–8.94)** | **0.01** |
| > 75% of LTL (longest) | 14/77 (18.2) | 1.11 (0.40–3.01) | 0.84 | 0.95 (0.34–2.63) | 0.92 |
| Linear trend (per 1,000 bp) | 66/243 (27.2) | 0.99 (0.69–1.44) | 0.97 | 0.93 (0.64–1.35) | 0.69 |
| LTL-PRS (per SD) | 66/243 (27.2) | 1.11 (0.86–1.44) | 0.42 | 1.14 (0.87–1.49) | 0.36 |
| **Male** |  |  |  |  |  |
| 0-25% of LTL (shortest) | 34/169 (20.1) | Ref. |  | Ref. |  |
| 25-50% of LTL | 33/179 (18.4) | 0.90 (0.53–1.53) | 0.69 | 0.80 (0.46–1.38) | 0.42 |
| 50-75% of LTL | 31/162 (19.1) | 0.94 (0.55–1.62) | 0.82 | 0.92 (0.53–1.60) | 0.76 |
| > 75% of LTL (longest) | 27/170 (15.9) | 0.75 (0.43–1.31) | 0.31 | 0.73 (0.41–1.30) | 0.28 |
| Linear trend (per 1,000 bp) | 125/680 (18.4) | 0.89 (0.70–1.15) | 0.37 | 0.90 (0.60–1.16) | 0.41 |
| LTL-PRS (per SD) | 125/680 (18.4) | 1.03 (0.84–1.26) | 0.79 | 1.04 (0.85–1.28) | 0.70 |

**Note:** Model 1 = unadjusted; Model 2 = adjusted for age, sex, and education. LTL-PRS is z-standardised within the analytic cohort; effects are per 1 SD. Linear trend for LTL is per 1,000 bp. Two-sided *p*-values are nominal (not adjusted for multiple testing).

**Supplementary Table S6. Association of leukocyte telomere length (LTL) and LTL polygenic risk score with post-stroke cognitive impairment stratified by TOAST subtype**

| **Exposure** | **Events n (%)** | **Model 1**  **OR (95% CI)** | ***P*** | **Model 2**  **OR (95% CI)** | ***P*** |
| --- | --- | --- | --- | --- | --- |
| **LAA** |  |  |  |  |  |
| 0-25% of LTL (shortest) | 13/61 (21.3) | Ref. |  | Ref. |  |
| 25-50% of LTL | 16/68 (23.5) | 1.14 (0.50–2.61) | 0.76 | 1.17 (0.50–2.75) | 0.72 |
| 50-75% of LTL | 12/52 (23.1) | 1.11 (0.46–2.70) | 0.82 | 1.13 (0.45–2.82) | 0.80 |
| > 75% of LTL (longest) | 10/50 (20.0) | 0.92 (0.37–2.33) | 0.87 | 0.87 (0.34–2.26) | 0.78 |
| Linear trend (per 1,000 bp) | 51/231 (22.1) | 1.01 (0.68–1.51) | 0.96 | 0.97 (0.64–1.47) | 0.88 |
| LTL-PRS (per SD) | 51/231 (22.1) | 0.76 (0.55–1.04) | 0.09 | **0.71 (0.51–0.98)** | **0.04** |
| **Cardioembolism** |  |  |  |  |  |
| 0-25% of LTL (shortest) | 3/9 (33.3) | Ref. |  | Ref. |  |
| 25-50% of LTL | 2/16 (12.5) | 0.29 (0.04–2.17) | 0.23 | 0.24 (0.03–2.02) | 0.19 |
| 50-75% of LTL | 9/21 (42.9) | 1.50 (0.29–7.68) | 0.63 | 2.05 (0.36–11.62) | 0.42 |
| > 75% of LTL (longest) | 1/10 (10.0) | 0.22 (0.02–2.67) | 0.24 | 0.21 (0.02–2.86) | 0.24 |
| Linear trend (per 1,000 bp) | 15/56 (26.8) | 0.64 (0.26–1.60) | 0.34 | 0.66 (0.26–1.65) | 0.37 |
| LTL-PRS (per SD) | 15/56 (26.8) | 0.91 (0.50–1.68) | 0.77 | 1.00 (0.52–1.95) | 0.99 |
| **SVO** |  |  |  |  |  |
| 0-25% of LTL (shortest) | 10/47 (21.3) | Ref. |  | Ref. |  |
| 25-50% of LTL | 9/43 (20.9) | 0.98 (0.36–2.70) | 0.97 | 0.92 (0.33–2.58) | 0.88 |
| 50-75% of LTL | 15/60 (25.0) | 1.23 (0.50–3.07) | 0.65 | 1.17 (0.46–2.94) | 0.75 |
| > 75% of LTL (longest) | 10/74 (13.5) | 0.58 (0.22–1.52) | 0.27 | 0.54 (0.20–1.45) | 0.22 |
| Linear trend (per 1,000 bp) | 44/224 (19.6) | 0.83 (0.55–1.25) | 0.37 | 0.82 (0.54–1.25) | 0.36 |
| LTL-PRS (per SD) | 44/224 (19.6) | **1.60 (1.13–2.28)** | **0.008** | **1.67 (1.16–2.39)** | **0.005** |
| **Others/Undet.** |  |  |  |  |  |
| 0-25% of LTL (shortest) | 15/94 (16.0) | Ref. |  | Ref. |  |
| 25-50% of LTL | 23/106 (21.7) | 1.46 (0.71–3.00) | 0.30 | 1.33 (0.64–2.76) | 0.44 |
| 50-75% of LTL | 23/99 (23.2) | 1.59 (0.77–3.28) | 0.21 | 1.48 (0.71–3.07) | 0.30 |
| > 75% of LTL (longest) | 20/113 (17.7) | 1.13 (0.54–2.36) | 0.74 | 1.07 (0.51–2.24) | 0.87 |
| Linear trend (per 1,000 bp) | 81/412 (19.7) | 1.06 (0.77–1.45) | 0.72 | 1.04 (0.75–1.44) | 0.82 |
| LTL-PRS (per SD) | 81/412 (19.7) | 1.02 (0.80–1.30) | 0.87 | 1.05 (0.82–1.34) | 0.72 |

**Note:** Model 1 = unadjusted; Model 2 = adjusted for age, sex, and education. LTL-PRS is z-standardised within the analytic cohort; effects are per 1 SD. Linear trend for LTL is per 1,000 bp. Two-sided *p*-values are nominal (no adjusted for multiple testing). TOAST subtypes were large-artery atherosclerosis, small-vessel occlusion, cardioembolism, and other determined/undetermined aetiology.

**Supplementary Table S7. Association between leukocyte telomere length and MoCA subdomain scores (ordinal logistic regression, unadjusted model)**

| **Exposure** | **Attention** | | **Executive** | | **Language** | | **Memory** | | **Visuospatial** | |
| --- | --- | --- | --- | --- | --- | --- | --- | --- | --- | --- |
|  | **cOR (95% CI)** | ***P*** | **cOR (95% CI)** | ***P*** | **cOR (95% CI)** | ***P*** | **cOR (95% CI)** | ***P*** | **cOR (95% CI)** | ***P*** |
| 0-25% of LTL (shortest) | Ref. |  | Ref. |  | Ref. |  | Ref. |  | Ref. |  |
| 25-50 % of LTL | 1.12 (0.77–1.62) | 0.49 | **1.45 (1.00–2.10)** | **0.02** | 1.17 (0.83–1.64) | 0.35 | **1.41 (1.02–1.94)** | **0.04** | 1.35 (0.93–1.96) | 0.09 |
| 50-75 % of LTL | 1.05 (0.73–1.53) | 0.87 | 1.15 (0.80–1.66) | 0.78 | 1.26 (0.89–1.77) | 0.10 | 1.27 (0.92–1.75) | 0.31 | 1.31 (0.90–1.90) | 0.16 |
| > 75 % of LTL (longest) | 0.97 (0.67–1.41) | 0.58 | 0.93 (0.64–1.34) | 0.11 | **0.86 (0.61–1.20)** | **0.04** | 0.98 (0.71–1.34) | 0.10 | **0.87 (0.60–1.28)** | **0.04** |
| Linear trend (per 1,000 bp) | 0.97 (0.82–1.15) | 0.73 | 0.94 (0.80–1.12) | 0.50 | 0.90 (0.78–1.05) | 0.19 | 0.98 (0.85–1.13) | 0.76 | 0.93 (0.79–1.11) | 0.42 |
| LTL-PRS (per SD) | 1.02 (0.90–1.16) | 0.74 | 1.11 (0.98–1.26) | 0.10 | 1.11 (0.99–1.25) | 0.08 | 1.01 (0.90–1.12) | 0.93 | 1.11 (0.98–1.26) | 0.11 |

Note. Estimates are common odds ratios (cORs) with 95% confidence intervals from proportional-odds (ordinal) logistic regression for higher MoCA subdomain scores. The proportional-odds assumption was assessed using a score test and was not violated for any sub-domain (all p > 0.05). Bold indicates nominal statistical significance (two-sided *p* < 0.05).

**Supplementary Table S8.** **Sensitivity analysis using an alternative PSCI definition (MoCA ≤22) (sample n = 974)**

| **Outcomes** | Events n (%) | Model 1  OR (95% CI) | P | Model 2  OR (95% CI) | P | Model 3  OR (95% CI) | P |
| --- | --- | --- | --- | --- | --- | --- | --- |
| **Telomere Length** |  |  |  |  |  |  |  |
| 0-25% of LTL (shortest) | 68/228 (29.8) | Ref. | Ref. | Ref. | Ref. | Ref. | Ref. |
| 25-50 % of LTL | 82/247 (33.2) | 1.17 (0.79-1.72) | 0.43 | 1.09 (0.73-1.62) | 0.68 | 1.07 (0.72-1.61) | 0.73 |
| 50-75 % of LTL | 90/243 (37.0) | 1.38 (0.94-2.03) | 0.10 | 1.37 (0.92-2.04) | 0.12 | 1.39 (0.93-2.07) | 0.11 |
| > 75 % of LTL (longest) | 79/256 (30.9) | 1.05 (0.71-1.55) | 0.80 | 1.06 (0.71-1.59) | 0.76 | 1.09 (0.73-1.63) | 0.69 |
| Linear trend (per 1000 bp) | 319/974 (32.8) | 0.999 (0.84-1.19) | 0.99 | 1.02 (0.85-1.22) | 0.85 | 1.03 (0.86-1.23) | 0.77 |
| LTL-PRS (per SD) | 319/974 (32.8) | 1.09 (0.95-1.25) | 0.20 | 1.11 (0.97-1.27) | 0.15 | 1.10 (0.96-1.27) | 0.17 |
| **Age subgroup analyses** |  |  |  |  |  |  |  |
| **Age ≤ 65 years old** |  |  |  |  |  |  |  |
| 0-25% of LTL (shortest) | 35/149 (23.5) | Ref. | Ref. | Ref. | Ref. | Ref. | Ref. |
| 25-50 % of LTL | 34/147 (23.1) | 0.98 (0.57-1.68) | 0.94 | 0.92 (0.53-1.60) | 0.77 | 0.91 (0.52-1.58) | 0.74 |
| 50-75 % of LTL | 55/155 (35.5) | **1.79 (1.09-2.96)** | **0.02** | **1.82 (1.09-3.04)** | **0.02** | **1.83 (1.10-3.07)** | **0.02** |
| > 75 % of LTL (longest) | 37/170 (21.8) | 0.91 (0.54-1.53) | 0.71 | 0.95 (0.55-1.63) | 0.85 | 0.96 (0.56-1.66) | 0.89 |
| Linear trend (per 1000 bp) | 161/621 (25.9) | 0.98 (0.79-1.22) | 0.84 | 1.01 (0.80-1.26) | 0.95 | 1.01 (0.81-1.27) | 0.91 |
| LTL-PRS (per SD) | 161/621 (25.9) | 1.10 (0.91-1.32) | 0.33 | 1.13 (0.93-1.36) | 0.21 | 1.14 (0.94-1.37) | 0.19 |
| **Age > 65 years old** |  |  |  |  |  |  |  |
| 0-25% of LTL (shortest) | 33/79 (41.8) | Ref. | Ref. | Ref. | Ref. | Ref. | Ref. |
| 25-50 % of LTL | 48/100 (48.0) | 1.29 (0.71-2.33) | 0.41 | 1.28 (0.70-2.33) | 0.43 | 1.27 (0.69-2.34) | 0.45 |
| 50-75 % of LTL | 35/88 (39.8) | 0.92 (0.50-1.71) | 0.79 | 0.87 (0.47-1.64) | 0.67 | 0.89 (0.47-1.67) | 0.71 |
| > 75 % of LTL (longest) | 42/86 (48.8) | 1.33 (0.72-2.46) | 0.36 | 1.24 (0.66-2.31) | 0.51 | 1.26 (0.67-2.37) | 0.48 |
| Linear trend (per 1000 bp) | 158/353 (44.8) | 1.08 (0.80-1.45) | 0.63 | 1.03 (0.76-1.40) | 0.84 | 1.04 (0.76-1.42) | 0.80 |
| LTL-PRS (per SD) | 158/353 (44.8) | 1.10 (0.90-1.36) | 0.34 | 1.09 (0.89-1.34) | 0.41 | 1.08 (0.87-1.33) | 0.50 |
| **Gender subgroup** |  |  |  |  |  |  |  |
| **Male** |  |  |  |  |  |  |  |
| 0-25% of LTL (shortest) | 53/183 (29.0) | Ref. | Ref. | Ref. | Ref. | Ref. | Ref. |
| 25-50 % of LTL | 60/190 (31.6) | 1.13 (0.73-1.76) | 0.58 | 1.06 (0.67-1.67) | 0.80 | 1.02 (0.65-1.62) | 0.92 |
| 50-75 % of LTL | 55/172 (32.0) | 1.15 (0.73-1.81) | 0.54 | 1.18 (0.74-1.87) | 0.49 | 1.16 (0.73-1.86) | 0.52 |
| > 75 % of LTL (longest) | 49/174 (28.2) | 0.96 (0.61-1.52) | 0.87 | 1.01 (0.63-1.62) | 0.98 | 1.02 (0.63-1.64) | 0.94 |
| Linear trend (per 1000 bp) | 217/719 (30.2) | 0.94 (0.77-1.16) | 0.57 | 0.98 (0.79-1.21) | 0.83 | 0.98 (0.79-1.22) | 0.87 |
| LTL-PRS (per SD) | 217/719 (30.2) | 1.11 (0.94-1.31) | 0.21 | 1.13 (0.95-1.34) | 0.17 | 1.13 (0.95-1.34) | 0.16 |
| **Female** |  |  |  |  |  |  |  |
| 0-25% of LTL (shortest) | 15/45 (33.3) | Ref. | Ref. | Ref. | Ref. | Ref. | Ref. |
| 25-50 % of LTL | 22/57 (38.6) | 1.26 (0.56-2.85) | 0.58 | 1.20 (0.52-2.79) | 0.66 | 1.46 (0.61-3.48) | 0.39 |
| 50-75 % of LTL | 35/71 (49.3) | 1.94 (0.90-4.22) | 0.09 | 2.11 (0.95-4.71) | 0.07 | 2.38 (1.05-5.41) | **0.04** |
| > 75 % of LTL (longest) | 30/82 (36.6) | 1.15 (0.54-2.48) | 0.71 | 1.28 (0.58-2.83) | 0.54 | 1.35 (0.60-3.03) | 0.46 |
| Linear trend (per 1000 bp) | 102/255 (40.0) | 1.05 (0.76-1.46) | 0.75 | 1.12 (0.80-1.58) | 0.50 | 1.12 (0.79-1.59) | 0.51 |
| LTL-PRS (per SD) | 102/255 (40.0) | 1.10 (0.87-1.39) | 0.43 | 1.07 (0.85-1.36) | 0.56 | 1.04 (0.82-1.33) | 0.74 |
| **TOAST subtype** |  |  |  |  |  |  |  |
| **Large-artery atherosclerosis** |  |  |  |  |  |  |  |
| 0-25% of LTL (shortest) | 19/63 (30.2) | Ref. | Ref. | Ref. | Ref. | Ref. | Ref. |
| 25-50 % of LTL | 31/74 (41.9) | 1.67 (0.82-3.39) | 0.16 | 1.63 (0.78-3.38) | 0.19 | 1.54 (0.73-3.21) | 0.25 |
| 50-75 % of LTL | 24/58 (41.4) | 1.64 (0.77-3.46) | 0.20 | 1.59 (0.73-3.45) | 0.24 | 1.56 (0.72-3.41) | 0.26 |
| > 75 % of LTL (longest) | 20/51 (39.2) | 1.49 (0.69-3.25) | 0.31 | 1.47 (0.66-3.27) | 0.35 | 1.45 (0.65-3.23) | 0.36 |
| Linear trend (per 1000 bp) | 94/246 (38.2) | 1.12 (0.80-1.57) | 0.51 | 1.10 (0.77-1.56) | 0.60 | 1.10 (0.78-1.57) | 0.58 |
| LTL-PRS (per SD) | 94/246 (38.2) | 1.09 (0.84-1.42) | 0.52 | 1.04 (0.79-1.36) | 0.78 | 1.07 (0.81-1.40) | 0.65 |
| **Cardioembolism** |  |  |  |  |  |  |  |
| 0-25% of LTL (shortest) | 4/9 (44.4) | Ref. | Ref. | Ref. | Ref. | Ref. | Ref. |
| 25-50 % of LTL | 4/19 (21.1) | 0.33 (0.06-1.85) | 0.21 | 0.37 (0.06-2.14) | 0.27 | 0.37 (0.07-2.16) | 0.27 |
| 50-75 % of LTL | 14/21 (66.7) | 2.50 (0.51-12.35) | 0.26 | 2.57 (0.50-13.24) | 0.26 | 3.55 (0.64-19.53) | 0.15 |
| > 75 % of LTL (longest) | 4/13 (30.8) | 0.56 (0.10-3.25) | 0.51 | 0.64 (0.10-4.05) | 0.64 | 0.63 (0.10-4.01) | 0.63 |
| Linear trend (per 1000 bp) | 26/62 (41.9) | 0.99 (0.47-2.09) | 0.97 | 0.95 (0.43-2.10) | 0.90 | 0.93 (0.42-2.07) | 0.86 |
| LTL-PRS (per SD) | 26/62 (41.9) | 0.93 (0.55-1.59) | 0.79 | 0.93 (0.53-1.61) | 0.79 | 0.92 (0.52-1.63) | 0.76 |
| **Small-vessel occlusion** |  |  |  |  |  |  |  |
| 0-25% of LTL (shortest) | 12/49 (24.5) | Ref. | Ref. | Ref. | Ref. | Ref. | Ref. |
| 25-50 % of LTL | 15/45 (33.3) | 1.54 (0.63-3.79) | 0.35 | 1.64 (0.63-4.28) | 0.31 | 1.59 (0.61-4.16) | 0.34 |
| 50-75 % of LTL | 18/62 (29.0) | 1.26 (0.54-2.96) | 0.59 | 1.23 (0.50-3.05) | 0.65 | 1.11 (0.44-2.79) | 0.83 |
| > 75 % of LTL (longest) | 20/76 (26.3) | 1.10 (0.48-2.52) | 0.82 | 1.23 (0.51-2.99) | 0.65 | 1.14 (0.46-2.80) | 0.78 |
| Linear trend (per 1000 bp) | 65/232 (28.0) | 0.92 (0.64-1.31) | 0.63 | 0.97 (0.65-1.45) | 0.90 | 0.93 (0.62-1.40) | 0.73 |
| LTL-PRS (per SD) | 65/232 (28.0) | 1.22 (0.91-1.62) | 0.18 | 1.28 (0.94-1.74) | 0.12 | 1.27 (0.93-1.73) | 0.13 |
| **Others and undetermined etiology** |  |  |  |  |  |  |  |
| 0-25% of LTL (shortest) | 33/107 (30.8) | Ref. | Ref. | Ref. | Ref. | Ref. | Ref. |
| 25-50 % of LTL | 32/109 (29.4) | 0.93 (0.52-1.67) | 0.81 | 0.86 (0.47-1.59) | 0.64 | 0.89 (0.48-1.65) | 0.71 |
| 50-75 % of LTL | 34/102 (33.3) | 1.12 (0.63-2.01) | 0.70 | 1.14 (0.62-2.10) | 0.68 | 1.21 (0.65-2.25) | 0.54 |
| > 75 % of LTL (longest) | 35/116 (30.2) | 0.97 (0.55-1.71) | 0.91 | 1.00 (0.55-1.83) | 0.99 | 1.03 (0.56-1.89) | 0.93 |
| Linear trend (per 1000 bp) | 134/434 (30.9) | 1.02 (0.78-1.32) | 0.91 | 1.06 (0.80-1.39) | 0.70 | 1.08 (0.82-1.43) | 0.58 |
| LTL-PRS (per SD) | 134/434 (30.9) | 1.05 (0.86-1.29) | 0.62 | 1.09 (0.88-1.35) | 0.42 | 1.08 (0.87-1.33) | 0.51 |

**Note:** PSCI was defined as MoCA ≤22 at 12 months. Odds ratios (ORs) and 95% confidence intervals (CIs) were estimated using logistic regression. Model 1 = unadjusted; Model 2 = adjusted for age, sex, and education; Model 3 = adjusted for age, sex, education, TIA history, NIHSS score at admission, TOAST classification. LTL was analysed in quartiles (Q1: 0-25% of LTL as reference) and as a continuous measure per 1,000 bp increase. LTL-PRS was z-standardised within the analytic cohort and modelled per SD increase. Two-sided *p* < 0.05 was considered statistically significant. Subgroup analyses were exploratory and not adjusted for multiple testing.

**Supplementary Table S9. Sensitivity analysis using an alternative PSCI definition (MoCA ≤24) (sample n = 974)**

| **Outcomes** | Events n (%) | Model 1  OR (95% CI) | P | Model 2  OR (95% CI) | P | Model 3  OR (95% CI) | P |
| --- | --- | --- | --- | --- | --- | --- | --- |
| **Telomere Length** |  |  |  |  |  |  |  |
| 0-25% of LTL (shortest) | 102/228 (44.7) | Ref. | Ref. | Ref. | Ref. | Ref. | Ref. |
| 25-50 % of LTL | 126/247 (51.0) | 1.29 (0.90-1.85) | 0.17 | 1.19 (0.82-1.73) | 0.36 | 1.19 (0.82-1.74) | 0.37 |
| 50-75 % of LTL | 116/243 (47.7) | 1.13 (0.79-1.62) | 0.51 | 1.09 (0.75-1.59) | 0.65 | 1.10 (0.75-1.61) | 0.63 |
| > 75 % of LTL (longest) | 113/256 (44.1) | 0.98 (0.68-1.40) | 0.90 | 0.99 (0.67-1.42) | 0.89 | 0.97 (0.66-1.41) | 0.86 |
| Linear trend (per 1000 bp) | 457/974 (46.9) | 0.97 (0.82-1.13) | 0.66 | 0.98 (0.83-1.15) | 0.79 | 0.98 (0.82-1.16) | 0.79 |
| LTL-PRS (per SD) | 457/974 (46.9) | 1.05 (0.92-1.19) | 0.48 | 1.07 (0.93-1.22) | 0.35 | 1.06 (0.93-1.21) | 0.36 |
| **Age subgroup** |  |  |  |  |  |  |  |
| **Age ≤ 65 years old** |  |  |  |  |  |  |  |
| 0-25% of LTL (shortest) | 53/149 (35.6) | Ref. | Ref. | Ref. | Ref. | Ref. | Ref. |
| 25-50 % of LTL | 64/147 (43.5) | 1.40 (0.88-2.23) | 0.16 | 1.31 (0.81-2.12) | 0.28 | 1.30 (0.80-2.11) | 0.29 |
| 50-75 % of LTL | 69/155 (44.5) | 1.45 (0.92-2.31) | 0.11 | 1.46 (0.91-2.36) | 0.12 | 1.45 (0.90-2.35) | 0.13 |
| > 75 % of LTL (longest) | 60/170 (35.3) | 0.99 (0.62-1.57) | 0.96 | 1.04 (0.64-1.67) | 0.89 | 1.03 (0.63-1.67) | 0.92 |
| Linear trend (per 1000 bp) | 246/621 (39.6) | 0.99 (0.82-1.21) | 0.94 | 1.03 (0.84-1.26) | 0.80 | 1.02 (0.83-1.26) | 0.83 |
| LTL-PRS (per SD) | 246/621 (39.6) | 1.00 (0.85-1.18) | 0.98 | 1.04 (0.88-1.23) | 0.67 | 1.04 (0.88-1.24) | 0.64 |
| **Age** > **65 years old** |  |  |  |  |  |  |  |
| 0-25% of LTL (shortest) | 49/79 (62.0) | Ref. | Ref. | Ref. | Ref. | Ref. | Ref. |
| 25-50 % of LTL | 62/100 (62.0) | 1.00 (0.54-1.84) | 0.99 | 0.96 (0.52-1.78) | 0.90 | 0.96 (0.52-1.79) | 0.90 |
| 50-75 % of LTL | 47/88 (53.4) | 0.70 (0.38-1.30) | 0.26 | 0.65 (0.34-1.21) | 0.17 | 0.64 (0.34-1.21) | 0.17 |
| > 75 % of LTL (longest) | 53/86 (61.6) | 0.98 (0.52-1.84) | 0.96 | 0.89 (0.47-1.68) | 0.71 | 0.89 (0.47-1.70) | 0.73 |
| Linear trend (per 1000 bp) | 211/353 (59.8) | 0.94 (0.69-1.27) | 0.66 | 0.88 (0.65-1.20) | 0.42 | 0.88 (0.64-1.20) | 0.42 |
| LTL-PRS (per SD) | 211/353 (59.8) | 1.14 (0.93-1.40) | 0.22 | 1.13 (0.91-1.39) | 0.26 | 1.04 (0.99-1.08) | 0.29 |
| **Gender subgroup** |  |  |  |  |  |  |  |
| **Male** |  |  |  |  |  |  |  |
| 0-25% of LTL (shortest) | 77/183 (42.1) | Ref. | Ref. | Ref. | Ref. | Ref. | Ref. |
| 25-50 % of LTL | 93/190 (49.0) | 1.32 (0.88-1.99) | 0.18 | 1.25 (0.82-1.90) | 0.31 | 1.24 (0.81-1.90) | 0.33 |
| 50-75 % of LTL | 72/172 (41.9) | 0.99 (0.65-1.51) | 0.97 | 1.01 (0.65-1.56) | 0.98 | 0.99 (0.64-1.53) | 0.95 |
| > 75 % of LTL (longest) | 71/174 (40.8) | 0.95 (0.62-1.45) | 0.81 | 1.01 (0.65-1.56) | 0.97 | 0.99 (0.64-1.54) | 0.97 |
| Linear trend (per 1000 bp) | 313/719 (43.5) | 0.92 (0.77-1.12) | 0.41 | 0.96 (0.79-1.18) | 0.72 | 0.96 (0.78-1.17) | 0.66 |
| LTL-PRS (per SD) | 313/719 (43.5) | 1.11 (0.95-1.30) | 0.18 | 1.13 (0.97-1.33) | 0.13 | 1.14 (0.97-1.34) | 0.11 |
| **Female** |  |  |  |  |  |  |  |
| 0-25% of LTL (shortest) | 25/45 (55.6) | Ref. | Ref. | Ref. | Ref. | Ref. | Ref. |
| 25-50 % of LTL | 33/57 (57.9) | 1.10 (0.50-2.42) | 0.81 | 1.04 (0.46-2.31) | 0.93 | 1.15 (0.51-2.61) | 0.74 |
| 50-75 % of LTL | 44/71 (62.0) | 1.30 (0.61-2.78) | 0.49 | 1.32 (0.61-2.86) | 0.48 | 1.40 (0.64-3.07) | 0.40 |
| > 75 % of LTL (longest) | 42/82 (51.2) | 0.84 (0.41-1.73) | 0.64 | 0.91 (0.43-1.91) | 0.80 | 0.93 (0.44-1.98) | 0.85 |
| Linear trend (per 1000 bp) | 144/255 (56.5) | 0.97 (0.70-1.34) | 0.83 | 1.01 (0.73-1.41) | 0.94 | 1.01 (0.72-1.41) | 0.97 |
| LTL-PRS (per SD) | 144/255 (56.5) | 0.97 (0.77-1.22) | 0.79 | 0.94 (0.74-1.19) | 0.61 | 0.92 (0.72-1.16) | 0.47 |
| **TOAST subtype** |  |  |  |  |  |  |  |
| **Large-artery atherosclerosis** |  |  |  |  |  |  |  |
| 0-25% of LTL (shortest) | 26/62 (41.3) | Ref. | Ref. | Ref. | Ref. | Ref. | Ref. |
| 25-50 % of LTL | 43/74 (58.1) | 1.97 (1.00-3.90) | **0.05** | 1.95 (0.96-3.97) | 0.06 | 1.89 (0.93-3.87) | 0.08 |
| 50-75 % of LTL | 26/58 (44.8) | 1.16 (0.56-2.38) | 0.69 | 1.13 (0.53-2.41) | 0.75 | 1.10 (0.51-2.36) | 0.80 |
| > 75 % of LTL (longest) | 25/51 (49.0) | 1.37 (0.65-2.88) | 0.41 | 1.36 (0.62-2.96) | 0.44 | 1.34 (0.62-2.93) | 0.46 |
| Linear trend (per 1000 bp) | 120/246 (48.8) | 1.08 (0.78-1.49) | 0.66 | 1.06 (0.75-1.50) | 0.75 | 1.06 (0.75-1.49) | 0.75 |
| LTL-PRS (per SD) | 120/246 (48.8) | 1.04 (0.81-1.34) | 0.75 | 0.98 (0.75-1.28) | 0.87 | 1.00 (0.76-1.30) | 0.97 |
| **Cardioembolism** |  |  |  |  |  |  |  |
| 0-25% of LTL (shortest) | 5/9 (55.6) | Ref. | Ref. | Ref. | Ref. | Ref. | Ref. |
| 25-50 % of LTL | 6/19 (31.6) | 0.37 (0.072-1.89) | 0.23 | 0.41 (0.08-2.19) | 0.30 | 0.41 (0.07-2.19) | 0.30 |
| 50-75 % of LTL | 16/21 (76.2) | 2.56 (0.49-13.30) | 0.27 | 2.58 (0.48-14.04) | 0.27 | 2.99 (0.52-17.21) | 0.22 |
| > 75 % of LTL (longest) | 8/13 (61.5) | 1.28 (0.23-7.19) | 0.78 | 1.52 (0.24-9.43) | 0.66 | 1.51 (0.24-9.43) | 0.66 |
| Linear trend (per 1000 bp) | 35/62 (56.5) | 1.59 (0.73-3.49) | 0.24 | 1.59 (0.68-3.70) | 0.28 | 1.60 (0.69-3.72) | 0.28 |
| LTL-PRS (per SD) | 35/62 (56.5) | 1.27 (0.74-2.18) | 0.38 | 1.31 (0.74-2.32) | 0.36 | 1.31 (0.73-2.35) | 0.37 |
| **Small-vessel occlusion** |  |  |  |  |  |  |  |
| 0-25% of LTL (shortest) | 19/49 (38.8) | Ref. | Ref. | Ref. | Ref. | Ref. | Ref. |
| 25-50 % of LTL | 23/45 (51.1) | 1.65 (0.73-3.75) | 0.23 | 1.67 (0.69-4.06) | 0.25 | 1.63 (0.67-3.98) | 0.29 |
| 50-75 % of LTL | 31/62 (50.0) | 1.58 (0.74-3.38) | 0.24 | 1.53 (0.68-3.47) | 0.31 | 1.29 (0.56-3.00) | 0.55 |
| > 75 % of LTL (longest) | 33/76 (43.4) | 1.21 (0.58-2.52) | 0.61 | 1.29 (0.58-2.87) | 0.53 | 1.15 (0.51-2.59) | 0.73 |
| Linear trend (per 1000 bp) | 106/232 (45.7) | 1.01 (0.73-1.39) | 0.98 | 1.07 (0.75-1.53) | 0.71 | 1.00 (0.70-1.44) | 0.99 |
| LTL-PRS (per SD) | 106/232 (45.7) | 1.10 (0.86-1.42) | 0.46 | 1.15 (0.87-1.51) | 0.32 | 1.13 (0.86-1.48) | 0.39 |
| **Others and undetermined etiology** |  |  |  |  |  |  |  |
| 0-25% of LTL (shortest) | 52/107 (48.6) | Ref. | Ref. | Ref. | Ref. | Ref. | Ref. |
| 25-50 % of LTL | 54/109 (49.5) | 1.04 (0.61-1.77) | 0.89 | 0.95 (0.54-1.66) | 0.85 | 0.99 (0.56-1.74) | 0.97 |
| 50-75 % of LTL | 43/102 (42.2) | 0.77 (0.45-1.33) | 0.35 | 0.75 (0.42-1.32) | 0.32 | 0.79 (0.45-1.41) | 0.43 |
| > 75 % of LTL (longest) | 47/116 (40.5) | 0.72 (0.42-1.22) | 0.23 | 0.72 (0.41-1.26) | 0.25 | 0.72 (0.41-1.27) | 0.26 |
| Linear trend (per 1000 bp) | 196/434 (45.2) | 0.85 (0.67-1.08) | 0.18 | 0.86 (0.67-1.11) | 0.25 | 0.88 (0.68-1.13) | 0.31 |
| LTL-PRS (per SD) | 196/434 (45.2) | 0.99 (0.82-1.19) | 0.89 | 1.02 (0.84-1.25) | 0.82 | 1.02 (0.84-1.25) | 0.82 |

**Note:** PSCI was defined as MoCA ≤24 at 12 months. Odds ratios (ORs) and 95% confidence intervals (CIs) were estimated using logistic regression. Model 1 = unadjusted; Model 2 = adjusted for age, sex, and education; Model 3 = adjusted for age, sex, education, TIA history, NIHSS score at admission, TOAST classification. LTL was analysed in quartiles (0-25% of LTL as reference) and as a continuous measure per 1,000 bp increase. LTL-PRS was z-standardised within the analytic cohort and modelled per SD increase. Two-sided *p* < 0.05 was considered statistically significant. Subgroup analyses were exploratory and not adjusted for multiple testing.
